# Syntheses, crystal structures and Hirshfeld surface analyses of (3a*R*,4*S*,7*R*,7a*S*)-2-(perfluoro­pyridin-4-yl)-3a,4,7,7a-tetra­hydro-4,7-methano­iso­indole-1,3-dione and (3a*R*,4*S*,7*R*,7a*S*)-2-[(perfluoro­pyridin-4-yl)­oxy]-3a,4,7,7a-tetra­hydro-4,7-methano­iso­indole-1,3-dione

**DOI:** 10.1107/S2056989019009769

**Published:** 2019-07-12

**Authors:** Andrew J. Peloquin, Gary J. Balaich, Scott T. Iacono

**Affiliations:** a Department of Chemistry & Chemistry Research Center, United States Air Force, Academy, Colorado Springs, CO 80840, USA

**Keywords:** crystal structure, perfluoro­pyridine, norbornene

## Abstract

The syntheses and crystal structures of the title compounds, C_14_H_8_F_4_N_2_O_2_ and C_14_H_8_F_4_N_2_O_3_, are reported. In each crystal, the packing is driven by C—H⋯F inter­tactions, along with a variety of C—H⋯O, C—O⋯π, and C—F⋯π contacts. Hirshfeld surface analysis was conducted to aid in the visualization of these various influences on the packing: they showed that the largest contributions to the surface contacts arise from H⋯F/F⋯H inter­actions, followed by H⋯H and O⋯H/H⋯O.

## Abstract 2

In each of the title compounds, the packing is driven by C—H⋯F inter­tactions, along with a variety of C—H⋯O, C—O⋯π, and C—F⋯π contacts. Hirshfeld surface analyses were conducted to aid in the visualization of these various influences on the packing.

## Chemical context   

Polynorbornenes (PNBs), derived from ring-opening metathesis polymerization reactions, are numerous, owing to their relative ease of synthesis, tolerance of diverse functional groups and high-mol­ecular weights with good processability (Isono *et al.*, 2018 ▸). The use of di­carb­oxy­imide-substituted norbornenes allows synthetic control of the substituents on the norbornene ring system, and this feature has been exploited for polymer light-emitting diodes (Zeng *et al.*, 2018 ▸) and gas-separation membranes (Yu *et al.*, 2016 ▸). With its predictable substitution chemistry (Baker & Muir, 2010 ▸; Chambers *et al.*, 1988 ▸), perfluoro­pyridine was added to two di­carb­oxy­imide-norbornene systems, and the resulting crystal structures are herein reported.
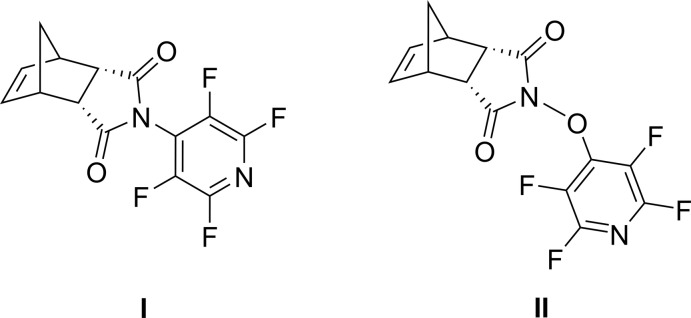


## Structural commentary   

**Figure 1:**
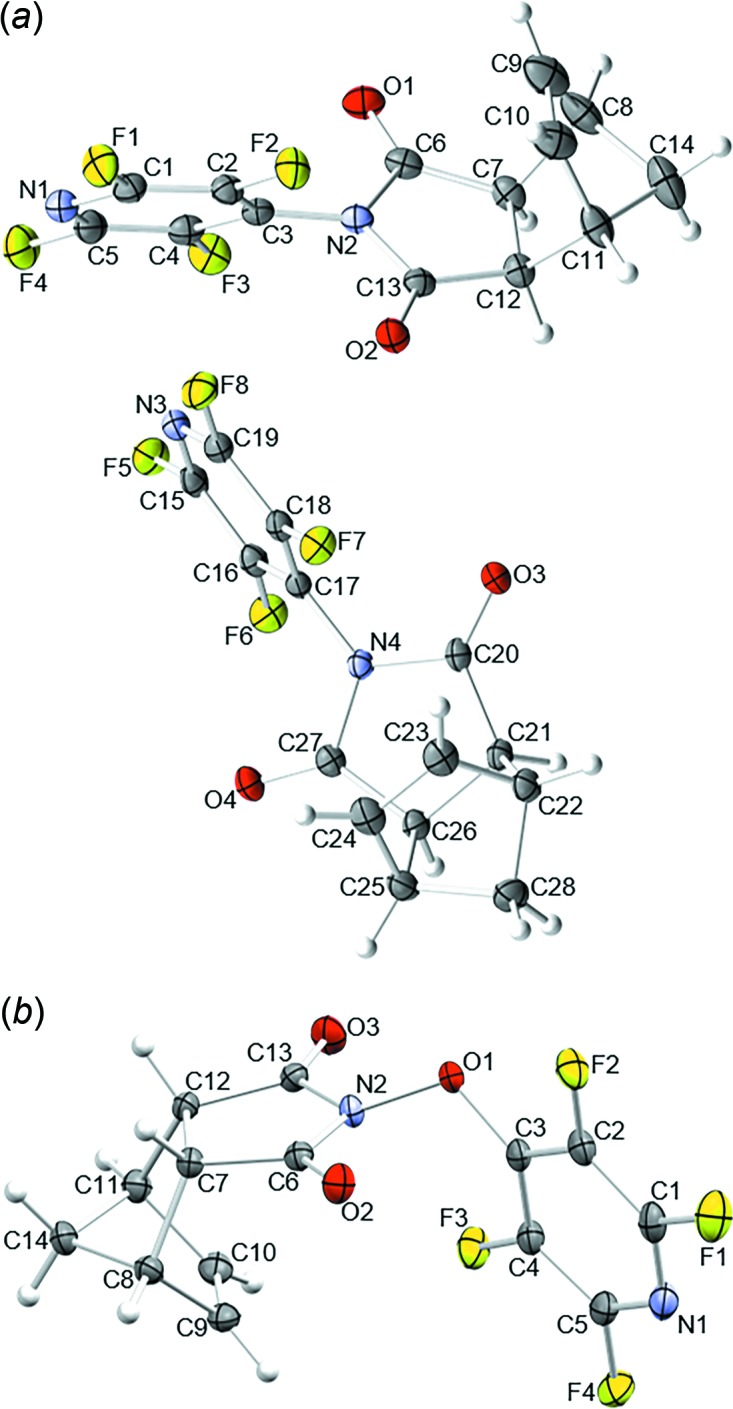
The mol­ecular structures of (*a*) **I** and (*b*) **II**. Displacement ellipsoids are shown at the 50% probability level.

Compound **I** crystallizes in the triclinic space group *P* 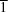 with two mol­ecules, *A* and *B*, per asymmetric unit, and compound **II** in the monoclinic space group *P*2_1_/*n* with one mol­ecule per asymmetric unit (Fig. 1 ▸). The synthesis of both compounds is conducted using *endo* starting materials, and the same configuration is observed in the resulting crystal structures. In **I**, steric inter­actions between the *ortho*-fluorine atoms and the carbonyl oxygen atoms prevents free rotation about the nitro­gen–*ipso-*carbon bond (C3—N2 and C17—N4 in the crystal): this is evidenced by separate ^19^F NMR peaks in solution for the *ortho-*F atoms (F2/F7 and F3/F6 in the crystal). In mol­ecule *A*, the N1/C1–C5 plane is rotated by 58.05 (5)° relative to the N2/C6/C7/C12/C13 plane and the corres­ponding dihedral angle for mol­ecule *B* is 61.65 (7)°. The addition of an oxygen atom between N2 and C3 in **II** alleviates this steric restriction and only one ^19^F NMR peak in solution is observed for the *ortho-*F atoms; even so, the dihedral angle between the N1/C1–C5 and N2/C6/C7/C12/C13 planes in the crystal of **II** of 84.01 (5)° is larger than those found in **I**.

## Supra­molecular features and Hirshfeld surface analysis   

The main directional inter­actions in the crystal structures of **I** and **II** are of the type C—H⋯O, C—H⋯F, C—O⋯π, and C—F⋯π (Tables 1 ▸ and 2 ▸). In both compounds, weak hydrogen-bonding inter­actions are observed for the hydrogen atom(s) α to the carbonyl groups (C7—H6⋯O1^i^, C12—H12⋯F2^iii^, C21—H21⋯F7^iii^, C21—H21⋯O3^iv^ and C26—H26⋯O4^v^ in **I**; C7—H7⋯O2^i^ in **II**) and the olefinic hydrogen atoms (C9—H9⋯F4^ii^ in **I**; C9—H9⋯O3^ii^ in **II**). A weak inter­action is also observed for a bridge hydrogen atom in **II**, C14—H14*B*⋯F4^iii^. The packing is further aided by π-inter­actions with the pyridine ring (C6—O1⋯*Cg* ^i^, C13—O2⋯*Cg* ^vi^ and C27—O4⋯*Cg* ^ii^ in **I**; C5—F4⋯*Cg* ^iv^ and C13—O3⋯*Cg* ^v^ in **II**).

**Figure 2:**
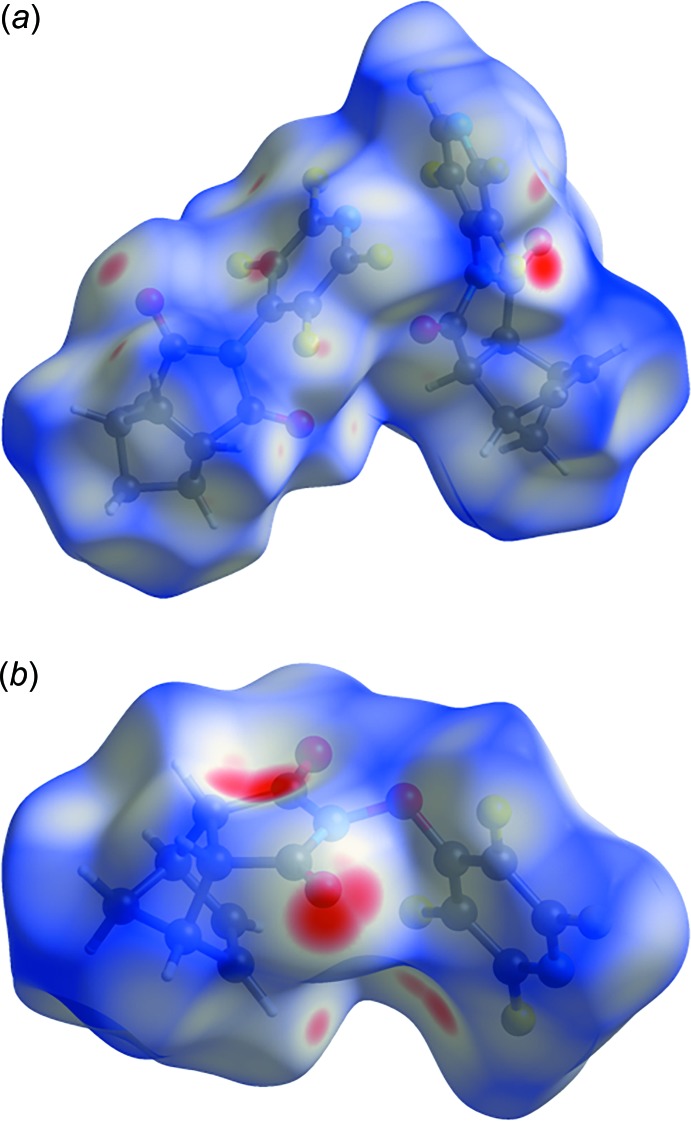
Hirshfeld surfaces of (*a*) **I** and (*b*) **II** mapped with *d*_norm_.

**Figure 3:**
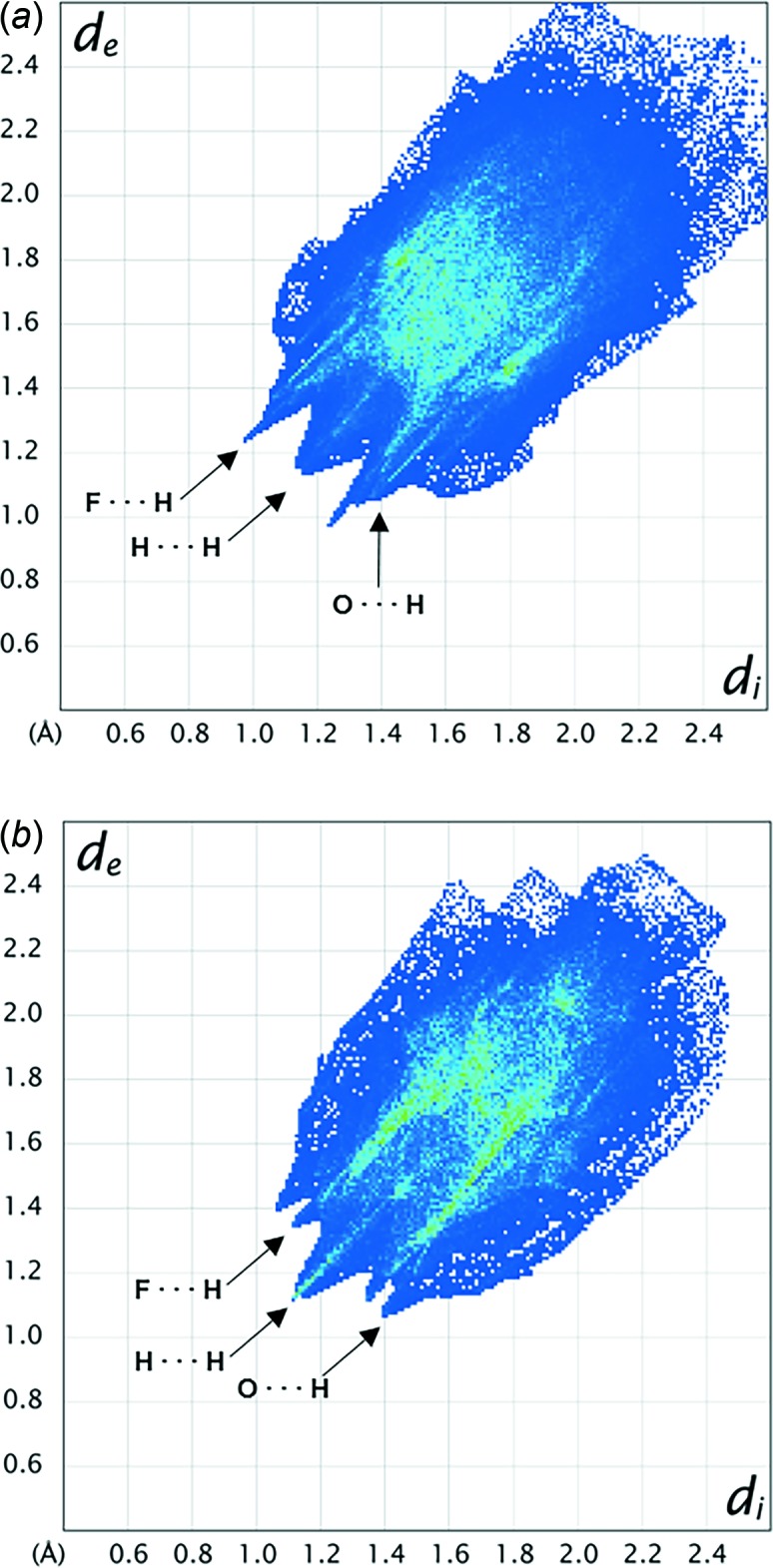
The overall two-dimensional fingerprint plots for (*a*) **I** and (*b*) **II**.

Hirshfeld surface analysis (Spackman & Jayatilaka, 2009 ▸) was used to investigate the presence of hydrogen bonds and other inter­molecular inter­actions in the crystal structures. The analyses and associated two-dimensional fingerprint plots (Fig. 3 ▸) (Spackman & McKinnon, 2002 ▸) were generated with *CrystalExplorer17.5* (Turner *et al.*, 2017 ▸) using a standard surface resolution with the three-dimensional *d*_norm_ surfaces plotted over a fixed color scale of −0.02500 (red) to 1.3800 (blue) a.u. The pale-red spots symbolize short contacts and negative *d*_norm_ values on the corresponding surface plots shown in Fig. 2 ▸, associated with their relative contributions to the Hirshfeld surface.

The largest contribution to the overall crystal packing in both compounds is from F⋯H/H⋯F inter­actions (36.5% in **I**; 39.2% in **II**; Table 3 ▸). The F⋯H/H⋯F contacts appear as a pair of characteristic tips in the fingerprint plots at 0.95 Å < (*d*_i_ + *d*_e_) < 1.25 Å in **I** and 1.10 Å < (*d*_i_ + *d*_e_) < 1.35 Å in **II**. H⋯H contacts make the second largest contribution (20.2% in **I** and 14.1% in **II**), shown in the middle region 1.10 Å < (*d*_i_ + *d*_e_) < 1.18 Å in **I** and **II**. The third largest contribution is from O⋯H/H⋯O contacts. In **I**, the corresponding spike is partially overlapped with the spike representing F⋯H/H⋯F contacts, appearing at 1.05 Å < (*d*_i_ + *d*_e_) < 1.40 Å. The O⋯H/H⋯O spike is clearly visible in the fingerprint plot of **II**, shown in the region of 1.10 Å < (*d*_i_ + *d*_e_) < 1.40 Å.

## Database survey   

A search of the November 2018 release of the Cambridge Structure Database (Groom *et al.*, 2016 ▸), with updates through May 2019, was performed using the program *ConQuest* (Bruno *et al.*, 2002 ▸). The search was limited to organic structures with *R* ≤ 0.1. A search for tetra­hydro-1*H*-4,7-methano­iso­indole-1,3(2*H*)-dione-based compounds with an aromatic substituent on the nitro­gen atom yielded 58 results. The dihedral angle between the aromatic ring plane and the succinimide plane is bimodally distributed between 43 and 90°, with peaks near 60 and 75°.

## Synthesis and crystallization   

**Synthesis of (I):** penta­fluoro­pyridine (2.68 ml, 24.5 mmol), (3a*R*,4*S*,7*R*,7a*S*)-3a,4,7,7a-tetra­hydro-1*H*-4,7-methano­iso­indole-1,3(2*H*)-dione (4.0 g, 24.5 mmol), and tri­ethyl­amine (8.56 ml, 61.2 mmol) were combined in DMF (150 ml). The resulting solution was stirred at room temperature for 24 h. Diethyl ether (150 ml) and saturated aqueous ammonium chloride (100 ml) were added and the biphasic solution stirred vigorously for 2 h. The organic layer was separated and the remaining aqueous portion extracted with diethyl ether (2 × 150 ml). The combined organic fractions were washed with water (2 × 1 l) and brine (2 × 300 ml), dried over MgSO_4_, and the solvent removed *via* rotary evaporation. The resulting off-white solid was dissolved in refluxing EtOH (20 ml) and cooled to 278 K for 12 h. Vacuum filtration, washing with cold EtOH (20 ml), and vacuum drying afforded the target compound as a white, crystalline solid (6.18 g, 81%). ^1^H NMR (500 MHz, CDCl_3_): 6.28 (*s*, 2H), 3.58 (*s*, 2H), 3.54 (*s*, 2H), 1.74 (*dd*, 2H, *J* = 96, 9.0 Hz).^19^F NMR (471 MHz, CDCl_3_): −90.5 (2F), −141.7 (1F), −143.1 (1F).

**Synthesis of (II):** to a stirred solution of potassium carbonate (1 *M*, 140 ml), (3a*R*,4*S*,7*R*,7a*S*)-2-hy­droxy-3a,4,7,7a-tetra­hydro-1*H*-4,7-methano­iso­indole-1,3(2*H*)-dione (10.0 g, 55.9 mmol), penta­fluoro­pyridine (6.1 ml, 56 mmol), and 140 ml of DMF were added. The resulting solution was stirred at room temperature for 24 h. Diethyl ether (150 ml) and saturated aqueous ammonium chloride (100 ml) were added and the biphasic solution stirred vigorously for 2 h. The organic layer was separated and the remaining aqueous portion extracted with diethyl ether (2 × 150 ml). The combined organic fractions were washed with water (2 × 1 l) and brine (2 × 300 ml), dried over MgSO_4_, and the solvent removed *via* rotary evaporation. The resulting off-white solid was dissolved in refluxing EtOH (50 ml) and cooled to 278 K for 12 h. Vacuum filtration, washing with cold EtOH (20 ml) and vacuum drying afforded the target compound as a white, crystalline solid (12.04 g, 66%). ^1^H NMR (500 MHz, CDCl_3_): 6.23 (*s*, 2H), 3.50 (*s*, 2H), 3.34 (*s*, 2H), 1.68 (*dd*, 2H, *J* = 142, 9.0 Hz).^19^F NMR (471 MHz, CDCl_3_): −87.4 (2F), −156.3 (2F).

## Refinement   

Crystal data, data collection and structure refinement details are summarized in Table 4 ▸. H atoms were positioned geometrically and refined using a riding model with C—H = 0.95–1.0Å and *U*_iso_(H) = 1.2*U*_eq_(C).

## Supplementary Material

Crystal structure: contains datablock(s) global, I, II. DOI: 10.1107/S2056989019009769/hb7832sup1.cif

Structure factors: contains datablock(s) I. DOI: 10.1107/S2056989019009769/hb7832Isup2.hkl

Structure factors: contains datablock(s) II. DOI: 10.1107/S2056989019009769/hb7832IIsup3.hkl

Supporting information file. DOI: 10.1107/S2056989019009769/hb7832Isup4.cml

Supporting information file. DOI: 10.1107/S2056989019009769/hb7832IIsup5.cml

CCDC references: 1879244, 1885019

Additional supporting information:  crystallographic information; 3D view; checkCIF report

## Figures and Tables

**Table 1:** Contact geometry (Å, °) for **I** *Cg*1 and *Cg*2 are the centroids of the N1/C1–C5 and N3/C15–C19 rings, respectively.

*Y—*X*⋯A*	*Y—*X**	*X⋯A*	*Y⋯A*	*Y—*X*⋯A*
C7—H7⋯O1^i^	1.00	2.58	3.347 (2)	133
C9—H9⋯F4^ii^	0.95	2.49	3.348(20	150
C12—H12⋯F2^iii^	1.00	2.30	3.1903 (18)	147
C21—H21⋯F7^iii^	1.00	2.55	3.3855 (17)	141
C21—H21.·O3^iv^	1.00	2.51	3.2764 (18)	133
C26—H26⋯O4^v^	1.00	2.51	3.3299 (18)	139
C6—O1⋯*Cg*1^i^	1.2075 (17)	3.0462 (14)	4.022 (16)	135.86 (10)
C13—O2⋯*Cg*2^vi^	1.2028 (16)	3.1839 (14)	4.0238 (17)	126.98 (9)
C27—O4⋯*Cg*2^ii^	1.2102 (16)	3.2978 (14)	4.0644 (16)	121.58 (9)

Symmetry codes: (i) 1 − *x*, 1 − *y*, 2 − *z*; (ii) −*x*, 1 − *y*, 2 − *z*; (iii) 1 + *x*, *y*, *z*; (iv) 2 − *x*, 1 − *y*, 1 − *z*; (v) 2 − *x*, 2 − *y*, 1 − *z*; (iv) *x*, *y*, *z*.

**Table 2:** Contact geometry (Å, °) for **II** *Cg*1 is the centroid of the N1/C1–C5 ring.

*Y—*X*⋯A*	*Y—*X**	*X⋯A*	*Y⋯A*	*Y—*X*⋯A*
C7—H7⋯O2^i^	1.00	2.58	3.347 (2)	133
C9—H9⋯O3^ii^	0.95	2.49	3.348(20	150
C14—H14*B*⋯F4^iii^	1.00	2.30	3.1903 (18)	147
C5—F4⋯*Cg*1^iv^	1.3234 (13)	3.1543 (18)	3.892 (2)	114.36 (7)
C13—O3⋯*Cg*1^v^	1.2060 (14)	3.3224 (19)	4.231 (2)	132.8 (8)

Symmetry codes: (i) 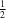 − *x*, 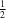 + *y*, 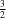 − *z*; (ii) *x*, −1 + *y*, *z*; (iii) 1 − *x*, 1 − *y*, 1 − *z*; (iv) 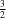 − *x*, −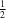 + *y*, 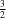 − *z*; (v) *x*, 1 + *y*, *z*.

**Table 3:** Percentage contribution of inter-atomic contacts to the Hirshfeld surfaces for **I** and **II**

Contact	Percentage contribution to **I**	Percentage contribution to **II**
F⋯H/H⋯F	36.5	39.2
H⋯H	20.2	14.1
O⋯H/H⋯O	13.0	14.0
O⋯C/C⋯O	6.2	6.2
N⋯H/H⋯N	5.3	0.1
F⋯F	5.1	2.1
F⋯C/C⋯F	3.6	4.9
F⋯N/N⋯F	2.4	4.4
O⋯N/N⋯O	0.6	4.1
C⋯H/H⋯C	3.8	3.2
F⋯O/O⋯F	2.4	2.7
O⋯O	1.2	2.7
N⋯C/C⋯N	0.0	2.4

**Table 4:** Experimental details

	**I**	**II**
Crystal data		
Chemical formula	C_14_H_8_F_4_N_2_O_2_	C_14_H_8_F_4_N_2_O_3_
*M*_r_	312.22	328.22
Crystal system, space group	Triclinic, *P* 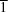	Monoclinic, *P*2_1_/*n*
Temperature (K)	100	100
*a*, *b*, *c* (Å)	7.0347 (15), 10.256 (2), 18.129 (4)	12.285 (6), 5.945 (3), 17.888 (8)
α, β, γ (°)	78.844 (10), 80.165 (10), 87.242 (10)	90, 93.44 (3), 90
*V* (Å^3^)	1264.2 (5)	1304.1 (10)
*Z*	4	4
Radiation type	Mo *K*α	Mo *K*α
μ (mm^−1^)	0.15	0.16
Crystal size (mm)	0.26 × 0.23 × 0.20	0.55 × 0.40 × 0.36

Data collection		
Diffractometer	Bruker SMART APEX CCD	Bruker SMART APEX CCD
Absorption correction	Multi-scan (*SADABS*; Bruker, 2017 ▸)	Multi-scan (*SADABS*; Bruker, 2017 ▸)
*T*_min_, *T*_max_	0.89, 0.97	0.80, 0.95
No. of measured, independent and observed [*I* > 2σ(*I*)] reflections	72226, 7674, 6373	18507, 3823, 3443
*R*_int_	0.034	0.025
(sin θ/λ)_max_ (Å^−1^)	0.714	0.704

Refinement		
*R*[*F* ^2^ > 2σ(*F* ^2^)], *wR*(*F* ^2^), *S*	0.045, 0.126, 1.02	0.038, 0.105, 1.04
No. of reflections	7674	3823
No. of parameters	397	208
H-atom treatment	H-atom parameters constrained	H-atom parameters constrained
Δρ_max_, Δρ_min_ (e Å^−3^)	1.77, −0.41	0.47, −0.25

Computer programs: *APEX3* and *SAINT* (Bruker, 2017 ▸), *SHELXT* (Sheldrick, 2015*a* ▸), *SHELXL2016* (Sheldrick, 2015*b* ▸), *Mercury* (Macrae *et al.*, 2008 ▸) and *publCIF* (Westrip, 2010 ▸).

## supplementary crystallographic information

### (I) Crystal data

**Table:**

C_14_H_8_F_4_N_2_O_2_	*Z* = 4
*M**_r_* = 312.22	*F*(000) = 632
Triclinic, *P*1	*D*_x_ = 1.640 Mg m^−^^3^
*a* = 7.0347 (15) Å	Mo *K*α radiation, λ = 0.71073 Å
*b* = 10.256 (2) Å	Cell parameters from 9823 reflections
*c* = 18.129 (4) Å	θ = 2.5–30.7°
α = 78.844 (10)°	µ = 0.15 mm^−^^1^
β = 80.165 (10)°	*T* = 100 K
γ = 87.242 (10)°	Rectangular prism, clear colourless
*V* = 1264.2 (5) Å^3^	0.26 × 0.23 × 0.20 mm

### (I) Data collection

**Table:**

Bruker SMART APEX CCD diffractometer	7674 independent reflections
Radiation source: fine focus sealed tube	6373 reflections with *I* > 2σ(*I*)
Graphite monochromator	*R*_int_ = 0.034
Detector resolution: 8.3333 pixels mm^-1^	θ_max_ = 30.5°, θ_min_ = 2.0°
ω Scans scans	*h* = −10→10
Absorption correction: multi-scan (SADABS; Bruker, 2017)	*k* = −14→14
*T*_min_ = 0.89, *T*_max_ = 0.97	*l* = −25→25
72226 measured reflections	

### (I) Refinement

**Table:**

Refinement on *F*^2^	Primary atom site location: structure-invariant direct methods
Least-squares matrix: full	Hydrogen site location: inferred from neighbouring sites
*R*[*F*^2^ > 2σ(*F*^2^)] = 0.045	H-atom parameters constrained
*wR*(*F*^2^) = 0.126	*w* = 1/[σ^2^(*F*_o_^2^) + (0.0625*P*)^2^ + 0.7892*P*] where *P* = (*F*_o_^2^ + 2*F*_c_^2^)/3
*S* = 1.02	(Δ/σ)_max_ = 0.001
7674 reflections	Δρ_max_ = 1.77 e Å^−^^3^
397 parameters	Δρ_min_ = −0.41 e Å^−^^3^
0 restraints	

### (I) Special details

**Table:**

Geometry. All esds (except the esd in the dihedral angle between two l.s. planes) are estimated using the full covariance matrix. The cell esds are taken into account individually in the estimation of esds in distances, angles and torsion angles; correlations between esds in cell parameters are only used when they are defined by crystal symmetry. An approximate (isotropic) treatment of cell esds is used for estimating esds involving l.s. planes.

### (I) Fractional atomic coordinates and isotropic or equivalent isotropic displacement parameters (Å^2^)

**Table:**

	*x*	*y*	*z*	*U*_iso_*/*U*_eq_	
F1	−0.32163 (13)	0.66535 (9)	0.80280 (6)	0.0299 (2)	
F2	−0.04925 (12)	0.46899 (8)	0.81310 (5)	0.02556 (18)	
F3	0.29897 (13)	0.74112 (9)	0.92385 (5)	0.02742 (19)	
F4	0.01062 (15)	0.92480 (9)	0.90912 (6)	0.0340 (2)	
F5	0.40224 (14)	0.92684 (9)	0.76901 (5)	0.02754 (19)	
F6	0.72380 (12)	0.90629 (9)	0.66496 (5)	0.02446 (18)	
F7	0.34503 (12)	0.67973 (9)	0.53929 (5)	0.02272 (17)	
F8	0.04320 (12)	0.70789 (9)	0.64982 (5)	0.02795 (19)	
O1	0.27798 (16)	0.45448 (11)	1.00080 (6)	0.0269 (2)	
O2	0.34535 (16)	0.50335 (10)	0.74150 (6)	0.0233 (2)	
O3	0.71312 (14)	0.54609 (9)	0.56707 (6)	0.02119 (19)	
O4	0.75913 (15)	0.99783 (9)	0.49291 (6)	0.02099 (19)	
N1	−0.15423 (18)	0.79402 (12)	0.85635 (7)	0.0235 (2)	
N2	0.28410 (16)	0.50134 (11)	0.87085 (6)	0.0178 (2)	
N3	0.22520 (17)	0.81750 (11)	0.70839 (7)	0.0208 (2)	
N4	0.70733 (15)	0.77544 (10)	0.54298 (6)	0.0154 (2)	
C1	−0.16515 (19)	0.68212 (14)	0.83262 (8)	0.0214 (3)	
C2	−0.02687 (19)	0.58195 (13)	0.83713 (8)	0.0192 (2)	
C3	0.13494 (18)	0.59829 (13)	0.86851 (7)	0.0172 (2)	
C4	0.1460 (2)	0.71642 (14)	0.89443 (7)	0.0204 (2)	
C5	−0.0016 (2)	0.80966 (14)	0.88622 (8)	0.0237 (3)	
C6	0.3481 (2)	0.43565 (14)	0.93807 (7)	0.0201 (2)	
C7	0.5095 (2)	0.34170 (14)	0.91564 (8)	0.0214 (3)	
H7	0.632591	0.364803	0.930317	0.026*	
C8	0.4631 (3)	0.19043 (16)	0.94528 (9)	0.0332 (3)	
H8	0.479722	0.15207	0.998913	0.04*	
C9	0.2739 (3)	0.16766 (17)	0.92242 (10)	0.0355 (4)	
H9	0.157787	0.146875	0.9574	0.043*	
C10	0.2883 (3)	0.17988 (17)	0.84872 (10)	0.0335 (3)	
H10	0.188245	0.170573	0.821019	0.04*	
C11	0.4917 (2)	0.21108 (14)	0.81733 (9)	0.0269 (3)	
H11	0.535408	0.194148	0.764515	0.032*	
C12	0.52885 (19)	0.35524 (13)	0.82814 (8)	0.0191 (2)	
H12	0.661556	0.384198	0.802588	0.023*	
C13	0.38067 (19)	0.45892 (13)	0.80420 (7)	0.0176 (2)	
C14	0.6008 (3)	0.13049 (17)	0.87797 (12)	0.0400 (4)	
H14A	0.737652	0.155649	0.871307	0.048*	
H14B	0.5897	0.033186	0.88305	0.048*	
C15	0.3933 (2)	0.86501 (13)	0.71103 (7)	0.0201 (2)	
C16	0.55752 (19)	0.85513 (13)	0.65845 (7)	0.0181 (2)	
C17	0.54593 (18)	0.79044 (12)	0.59868 (7)	0.0157 (2)	
C18	0.36863 (19)	0.73991 (12)	0.59592 (7)	0.0169 (2)	
C19	0.21476 (19)	0.75621 (13)	0.65218 (8)	0.0195 (2)	
C20	0.77807 (18)	0.64904 (12)	0.52953 (7)	0.0164 (2)	
C21	0.93999 (18)	0.67181 (12)	0.46283 (7)	0.0167 (2)	
H21	1.0639	0.631685	0.477318	0.02*	
C22	0.8943 (2)	0.62467 (13)	0.39011 (7)	0.0197 (2)	
H22	0.919783	0.528529	0.388159	0.024*	
C23	0.6930 (2)	0.67662 (14)	0.38012 (8)	0.0225 (3)	
H23	0.580506	0.624899	0.387553	0.027*	
C24	0.7034 (2)	0.80880 (14)	0.35888 (8)	0.0235 (3)	
H24	0.598626	0.867847	0.348918	0.028*	
C25	0.9114 (2)	0.84821 (13)	0.35344 (8)	0.0223 (3)	
H25	0.950085	0.936047	0.320653	0.027*	
C26	0.95440 (19)	0.82471 (12)	0.43732 (7)	0.0176 (2)	
H26	1.085803	0.856148	0.438844	0.021*	
C27	0.80172 (18)	0.88246 (12)	0.49164 (7)	0.0164 (2)	
C28	1.0183 (2)	0.72422 (15)	0.32846 (8)	0.0263 (3)	
H28A	1.004935	0.715936	0.276088	0.032*	
H28B	1.156118	0.719809	0.334083	0.032*	

### (I) Atomic displacement parameters (Å^2^)

**Table:**

	*U*^11^	*U*^22^	*U*^33^	*U*^12^	*U*^13^	*U*^23^
F1	0.0193 (4)	0.0288 (5)	0.0432 (5)	−0.0001 (3)	−0.0118 (4)	−0.0050 (4)
F2	0.0233 (4)	0.0211 (4)	0.0368 (5)	−0.0025 (3)	−0.0098 (3)	−0.0115 (3)
F3	0.0301 (5)	0.0295 (4)	0.0273 (4)	−0.0059 (4)	−0.0112 (4)	−0.0099 (4)
F4	0.0406 (5)	0.0235 (4)	0.0406 (5)	−0.0017 (4)	−0.0029 (4)	−0.0158 (4)
F5	0.0396 (5)	0.0255 (4)	0.0190 (4)	−0.0009 (4)	−0.0005 (3)	−0.0116 (3)
F6	0.0252 (4)	0.0264 (4)	0.0253 (4)	−0.0041 (3)	−0.0062 (3)	−0.0109 (3)
F7	0.0219 (4)	0.0269 (4)	0.0230 (4)	−0.0013 (3)	−0.0059 (3)	−0.0114 (3)
F8	0.0181 (4)	0.0307 (5)	0.0352 (5)	−0.0019 (3)	−0.0016 (3)	−0.0086 (4)
O1	0.0308 (5)	0.0333 (6)	0.0160 (4)	−0.0034 (4)	−0.0036 (4)	−0.0031 (4)
O2	0.0312 (5)	0.0217 (5)	0.0169 (4)	0.0030 (4)	−0.0050 (4)	−0.0036 (4)
O3	0.0229 (5)	0.0152 (4)	0.0238 (5)	0.0005 (3)	−0.0014 (4)	−0.0020 (4)
O4	0.0255 (5)	0.0145 (4)	0.0242 (5)	0.0000 (3)	−0.0055 (4)	−0.0054 (4)
N1	0.0226 (6)	0.0203 (5)	0.0254 (6)	−0.0001 (4)	0.0005 (4)	−0.0031 (4)
N2	0.0188 (5)	0.0197 (5)	0.0148 (5)	−0.0010 (4)	−0.0036 (4)	−0.0025 (4)
N3	0.0246 (6)	0.0153 (5)	0.0201 (5)	0.0027 (4)	0.0003 (4)	−0.0017 (4)
N4	0.0174 (5)	0.0132 (4)	0.0161 (5)	0.0007 (4)	−0.0024 (4)	−0.0043 (4)
C1	0.0169 (6)	0.0224 (6)	0.0241 (6)	−0.0026 (5)	−0.0020 (5)	−0.0027 (5)
C2	0.0194 (6)	0.0184 (6)	0.0202 (6)	−0.0034 (4)	−0.0024 (5)	−0.0049 (5)
C3	0.0173 (6)	0.0189 (6)	0.0147 (5)	−0.0018 (4)	−0.0013 (4)	−0.0021 (4)
C4	0.0220 (6)	0.0234 (6)	0.0166 (5)	−0.0042 (5)	−0.0025 (5)	−0.0053 (5)
C5	0.0276 (7)	0.0197 (6)	0.0236 (6)	−0.0025 (5)	0.0008 (5)	−0.0075 (5)
C6	0.0221 (6)	0.0218 (6)	0.0172 (5)	−0.0057 (5)	−0.0063 (5)	−0.0012 (5)
C7	0.0242 (6)	0.0210 (6)	0.0207 (6)	−0.0008 (5)	−0.0099 (5)	−0.0024 (5)
C8	0.0500 (10)	0.0231 (7)	0.0274 (7)	−0.0034 (6)	−0.0171 (7)	0.0037 (6)
C9	0.0504 (10)	0.0253 (7)	0.0336 (8)	−0.0101 (7)	−0.0105 (7)	−0.0069 (6)
C10	0.0407 (9)	0.0289 (8)	0.0327 (8)	−0.0103 (7)	−0.0069 (7)	−0.0072 (6)
C11	0.0374 (8)	0.0175 (6)	0.0303 (7)	0.0006 (5)	−0.0164 (6)	−0.0064 (5)
C12	0.0197 (6)	0.0179 (6)	0.0213 (6)	−0.0004 (4)	−0.0067 (5)	−0.0048 (5)
C13	0.0194 (6)	0.0167 (5)	0.0172 (5)	−0.0037 (4)	−0.0024 (4)	−0.0039 (4)
C14	0.0480 (10)	0.0213 (7)	0.0546 (11)	0.0049 (7)	−0.0255 (9)	−0.0033 (7)
C15	0.0297 (7)	0.0146 (5)	0.0160 (5)	0.0019 (5)	−0.0026 (5)	−0.0047 (4)
C16	0.0220 (6)	0.0155 (5)	0.0181 (5)	0.0002 (4)	−0.0055 (5)	−0.0042 (4)
C17	0.0188 (6)	0.0128 (5)	0.0151 (5)	0.0021 (4)	−0.0029 (4)	−0.0025 (4)
C18	0.0201 (6)	0.0147 (5)	0.0168 (5)	0.0020 (4)	−0.0047 (4)	−0.0041 (4)
C19	0.0191 (6)	0.0163 (5)	0.0220 (6)	0.0010 (4)	−0.0027 (5)	−0.0018 (5)
C20	0.0176 (5)	0.0149 (5)	0.0184 (5)	0.0019 (4)	−0.0059 (4)	−0.0051 (4)
C21	0.0168 (5)	0.0147 (5)	0.0195 (5)	0.0010 (4)	−0.0029 (4)	−0.0059 (4)
C22	0.0254 (6)	0.0161 (5)	0.0187 (6)	0.0002 (5)	−0.0029 (5)	−0.0065 (5)
C23	0.0275 (7)	0.0226 (6)	0.0204 (6)	−0.0013 (5)	−0.0081 (5)	−0.0079 (5)
C24	0.0314 (7)	0.0227 (6)	0.0187 (6)	0.0019 (5)	−0.0098 (5)	−0.0052 (5)
C25	0.0305 (7)	0.0170 (6)	0.0176 (6)	−0.0016 (5)	−0.0002 (5)	−0.0021 (5)
C26	0.0188 (6)	0.0148 (5)	0.0195 (6)	−0.0010 (4)	−0.0018 (4)	−0.0050 (4)
C27	0.0184 (5)	0.0158 (5)	0.0167 (5)	−0.0011 (4)	−0.0056 (4)	−0.0047 (4)
C28	0.0332 (8)	0.0245 (7)	0.0199 (6)	−0.0012 (6)	0.0023 (5)	−0.0069 (5)

### (I) Geometric parameters (Å, º)

**Table:**

F1—C1	1.3384 (16)	C9—C10	1.304 (2)
F2—C2	1.3390 (15)	C9—H9	0.95
F3—C4	1.3343 (16)	C10—C11	1.473 (2)
F4—C5	1.3364 (16)	C10—H10	0.95
F5—C15	1.3398 (15)	C11—C14	1.535 (2)
F6—C16	1.3370 (15)	C11—C12	1.5683 (19)
F7—C18	1.3325 (14)	C11—H11	1.0
F8—C19	1.3370 (16)	C12—C13	1.5084 (18)
O1—C6	1.2075 (17)	C12—H12	1.0
O2—C13	1.2028 (16)	C14—H14A	0.99
O3—C20	1.2038 (16)	C14—H14B	0.99
O4—C27	1.2102 (16)	C15—C16	1.3794 (19)
N1—C1	1.3111 (18)	C16—C17	1.3909 (17)
N1—C5	1.312 (2)	C17—C18	1.3858 (18)
N2—C6	1.4077 (17)	C18—C19	1.3825 (18)
N2—C3	1.4107 (17)	C20—C21	1.5033 (18)
N2—C13	1.4161 (17)	C21—C26	1.5499 (18)
N3—C19	1.3103 (18)	C21—C22	1.5753 (18)
N3—C15	1.3125 (19)	C21—H21	1.0
N4—C27	1.4056 (17)	C22—C23	1.516 (2)
N4—C17	1.4086 (16)	C22—C28	1.537 (2)
N4—C20	1.4165 (16)	C22—H22	1.0
C1—C2	1.3809 (19)	C23—C24	1.338 (2)
C2—C3	1.3871 (18)	C23—H23	0.95
C3—C4	1.3917 (18)	C24—C25	1.519 (2)
C4—C5	1.385 (2)	C24—H24	0.95
C6—C7	1.507 (2)	C25—C28	1.550 (2)
C7—C12	1.5482 (19)	C25—C26	1.5717 (19)
C7—C8	1.571 (2)	C25—H25	1.0
C7—H7	1.0	C26—C27	1.5091 (18)
C8—C9	1.501 (3)	C26—H26	1.0
C8—C14	1.623 (3)	C28—H28A	0.99
C8—H8	1.0	C28—H28B	0.99
			
C1—N1—C5	116.94 (12)	C8—C14—H14A	113.4
C6—N2—C3	124.33 (11)	C11—C14—H14B	113.4
C6—N2—C13	113.71 (11)	C8—C14—H14B	113.4
C3—N2—C13	121.94 (11)	H14A—C14—H14B	110.7
C19—N3—C15	117.26 (12)	N3—C15—F5	116.65 (12)
C27—N4—C17	123.69 (10)	N3—C15—C16	124.23 (12)
C27—N4—C20	113.87 (11)	F5—C15—C16	119.11 (12)
C17—N4—C20	122.21 (11)	F6—C16—C15	120.74 (11)
N1—C1—F1	117.06 (12)	F6—C16—C17	120.86 (12)
N1—C1—C2	124.07 (13)	C15—C16—C17	118.40 (12)
F1—C1—C2	118.87 (12)	C18—C17—C16	117.45 (12)
F2—C2—C1	120.87 (12)	C18—C17—N4	120.70 (11)
F2—C2—C3	119.96 (12)	C16—C17—N4	121.85 (12)
C1—C2—C3	119.15 (12)	F7—C18—C19	120.46 (12)
C2—C3—C4	116.92 (12)	F7—C18—C17	120.92 (11)
C2—C3—N2	121.00 (11)	C19—C18—C17	118.60 (12)
C4—C3—N2	122.02 (12)	N3—C19—F8	117.01 (12)
F3—C4—C5	120.87 (12)	N3—C19—C18	124.05 (13)
F3—C4—C3	120.66 (12)	F8—C19—C18	118.94 (12)
C5—C4—C3	118.42 (12)	O3—C20—N4	123.24 (12)
N1—C5—F4	116.75 (13)	O3—C20—C21	129.39 (12)
N1—C5—C4	124.49 (13)	N4—C20—C21	107.37 (10)
F4—C5—C4	118.75 (13)	C20—C21—C26	105.67 (10)
O1—C6—N2	123.41 (13)	C20—C21—C22	113.60 (11)
O1—C6—C7	129.08 (13)	C26—C21—C22	103.31 (10)
N2—C6—C7	107.50 (11)	C20—C21—H21	111.3
C6—C7—C12	105.79 (11)	C26—C21—H21	111.3
C6—C7—C8	114.60 (13)	C22—C21—H21	111.3
C12—C7—C8	102.66 (11)	C23—C22—C28	100.97 (11)
C6—C7—H7	111.1	C23—C22—C21	106.32 (10)
C12—C7—H7	111.1	C28—C22—C21	98.84 (11)
C8—C7—H7	111.1	C23—C22—H22	116.1
C9—C8—C7	106.69 (13)	C28—C22—H22	116.1
C9—C8—C14	97.02 (13)	C21—C22—H22	116.1
C7—C8—C14	98.55 (13)	C24—C23—C22	107.28 (13)
C9—C8—H8	117.1	C24—C23—H23	126.4
C7—C8—H8	117.1	C22—C23—H23	126.4
C14—C8—H8	117.1	C23—C24—C25	108.23 (13)
C10—C9—C8	111.81 (17)	C23—C24—H24	125.9
C10—C9—H9	124.1	C25—C24—H24	125.9
C8—C9—H9	124.1	C24—C25—C28	100.25 (11)
C9—C10—C11	105.78 (16)	C24—C25—C26	106.61 (11)
C9—C10—H10	127.1	C28—C25—C26	98.57 (11)
C11—C10—H10	127.1	C24—C25—H25	116.3
C10—C11—C14	103.19 (15)	C28—C25—H25	116.3
C10—C11—C12	107.98 (13)	C26—C25—H25	116.3
C14—C11—C12	99.67 (12)	C27—C26—C21	105.73 (10)
C10—C11—H11	114.8	C27—C26—C25	113.65 (11)
C14—C11—H11	114.8	C21—C26—C25	102.89 (10)
C12—C11—H11	114.8	C27—C26—H26	111.4
C13—C12—C7	105.61 (11)	C21—C26—H26	111.4
C13—C12—C11	115.44 (11)	C25—C26—H26	111.4
C7—C12—C11	103.66 (11)	O4—C27—N4	123.64 (12)
C13—C12—H12	110.6	O4—C27—C26	128.99 (12)
C7—C12—H12	110.6	N4—C27—C26	107.35 (10)
C11—C12—H12	110.6	C22—C28—C25	94.29 (11)
O2—C13—N2	123.30 (12)	C22—C28—H28A	112.9
O2—C13—C12	129.34 (12)	C25—C28—H28A	112.9
N2—C13—C12	107.35 (11)	C22—C28—H28B	112.9
C11—C14—C8	91.50 (13)	C25—C28—H28B	112.9
C11—C14—H14A	113.4	H28A—C28—H28B	110.3
			
C5—N1—C1—F1	179.36 (12)	C19—N3—C15—F5	−179.67 (11)
C5—N1—C1—C2	−0.2 (2)	C19—N3—C15—C16	0.4 (2)
N1—C1—C2—F2	178.68 (13)	N3—C15—C16—F6	179.92 (12)
F1—C1—C2—F2	−0.9 (2)	F5—C15—C16—F6	0.03 (19)
N1—C1—C2—C3	0.0 (2)	N3—C15—C16—C17	−0.2 (2)
F1—C1—C2—C3	−179.51 (12)	F5—C15—C16—C17	179.89 (11)
F2—C2—C3—C4	−178.07 (11)	F6—C16—C17—C18	179.90 (11)
C1—C2—C3—C4	0.57 (19)	C15—C16—C17—C18	0.04 (18)
F2—C2—C3—N2	4.45 (19)	F6—C16—C17—N4	0.29 (19)
C1—C2—C3—N2	−176.90 (12)	C15—C16—C17—N4	−179.57 (11)
C6—N2—C3—C2	−122.28 (14)	C27—N4—C17—C18	115.77 (14)
C13—N2—C3—C2	56.10 (17)	C20—N4—C17—C18	−58.41 (16)
C6—N2—C3—C4	60.38 (18)	C27—N4—C17—C16	−64.63 (17)
C13—N2—C3—C4	−121.24 (14)	C20—N4—C17—C16	121.18 (13)
C2—C3—C4—F3	−178.53 (12)	C16—C17—C18—F7	178.24 (11)
N2—C3—C4—F3	−1.08 (19)	N4—C17—C18—F7	−2.15 (18)
C2—C3—C4—C5	−1.01 (19)	C16—C17—C18—C19	−0.10 (18)
N2—C3—C4—C5	176.44 (12)	N4—C17—C18—C19	179.52 (11)
C1—N1—C5—F4	178.87 (12)	C15—N3—C19—F8	179.93 (11)
C1—N1—C5—C4	−0.3 (2)	C15—N3—C19—C18	−0.5 (2)
F3—C4—C5—N1	178.43 (13)	F7—C18—C19—N3	−178.00 (12)
C3—C4—C5—N1	0.9 (2)	C17—C18—C19—N3	0.3 (2)
F3—C4—C5—F4	−0.7 (2)	F7—C18—C19—F8	1.56 (19)
C3—C4—C5—F4	−178.23 (12)	C17—C18—C19—F8	179.90 (11)
C3—N2—C6—O1	1.5 (2)	C27—N4—C20—O3	−178.97 (12)
C13—N2—C6—O1	−176.98 (13)	C17—N4—C20—O3	−4.26 (19)
C3—N2—C6—C7	−179.43 (11)	C27—N4—C20—C21	1.03 (14)
C13—N2—C6—C7	2.06 (15)	C17—N4—C20—C21	175.74 (11)
O1—C6—C7—C12	176.95 (14)	O3—C20—C21—C26	178.83 (13)
N2—C6—C7—C12	−2.02 (14)	N4—C20—C21—C26	−1.17 (13)
O1—C6—C7—C8	64.62 (19)	O3—C20—C21—C22	66.28 (17)
N2—C6—C7—C8	−114.36 (13)	N4—C20—C21—C22	−113.72 (11)
C6—C7—C8—C9	51.32 (17)	C20—C21—C22—C23	46.18 (14)
C12—C7—C8—C9	−62.86 (16)	C26—C21—C22—C23	−67.79 (13)
C6—C7—C8—C14	151.35 (12)	C20—C21—C22—C28	150.43 (11)
C12—C7—C8—C14	37.17 (14)	C26—C21—C22—C28	36.46 (12)
C7—C8—C9—C10	68.24 (19)	C28—C22—C23—C24	−33.15 (14)
C14—C8—C9—C10	−32.91 (18)	C21—C22—C23—C24	69.55 (14)
C8—C9—C10—C11	0.42 (19)	C22—C23—C24—C25	0.53 (15)
C9—C10—C11—C14	35.12 (18)	C23—C24—C25—C28	31.90 (14)
C9—C10—C11—C12	−69.79 (17)	C23—C24—C25—C26	−70.35 (14)
C6—C7—C12—C13	1.33 (14)	C20—C21—C26—C27	0.93 (13)
C8—C7—C12—C13	121.79 (12)	C22—C21—C26—C27	120.51 (11)
C6—C7—C12—C11	−120.46 (12)	C20—C21—C26—C25	−118.55 (11)
C8—C7—C12—C11	−0.01 (14)	C22—C21—C26—C25	1.04 (12)
C10—C11—C12—C13	−47.45 (16)	C24—C25—C26—C27	−48.17 (14)
C14—C11—C12—C13	−154.82 (14)	C28—C25—C26—C27	−151.64 (11)
C10—C11—C12—C7	67.53 (14)	C24—C25—C26—C21	65.64 (12)
C14—C11—C12—C7	−39.85 (15)	C28—C25—C26—C21	−37.82 (12)
C6—N2—C13—O2	179.80 (12)	C17—N4—C27—O4	3.78 (19)
C3—N2—C13—O2	1.3 (2)	C20—N4—C27—O4	178.40 (12)
C6—N2—C13—C12	−1.18 (15)	C17—N4—C27—C26	−175.04 (11)
C3—N2—C13—C12	−179.73 (11)	C20—N4—C27—C26	−0.41 (14)
C7—C12—C13—O2	178.76 (14)	C21—C26—C27—O4	−179.08 (13)
C11—C12—C13—O2	−67.39 (19)	C25—C26—C27—O4	−66.97 (17)
C7—C12—C13—N2	−0.18 (14)	C21—C26—C27—N4	−0.35 (13)
C11—C12—C13—N2	113.68 (13)	C25—C26—C27—N4	111.75 (12)
C10—C11—C14—C8	−50.81 (14)	C23—C22—C28—C25	49.19 (12)
C12—C11—C14—C8	60.38 (14)	C21—C22—C28—C25	−59.46 (12)
C9—C8—C14—C11	47.84 (14)	C24—C25—C28—C22	−48.49 (12)
C7—C8—C14—C11	−60.29 (13)	C26—C25—C28—C22	60.24 (12)

### (I) Hydrogen-bond geometry (Å, º)

**Table:**

*D*—H···*A*	*D*—H	H···*A*	*D*···*A*	*D*—H···*A*
C7—H7···O1^i^	1.00	2.58	3.3467 (18)	133
C12—H12···F2^ii^	1.00	2.30	3.1902 (17)	147
C21—H21···F7^ii^	1.00	2.55	3.3855 (16)	141
C21—H21···O3^iii^	1.00	2.51	3.2765 (16)	133
C26—H26···O4^iv^	1.00	2.51	3.3299 (17)	139
C6—O1···*Cg*1^v^		3.05 (1)	4.0022 (16)	136 (1)
C13—O2···*Cg*2		3.18 (1)	4.0238 (17)	127 (1)
C27—O4···*Cg*2^vi^		3.30 (1)	4.0644 (16)	122 (1)

Symmetry codes: (i) −*x*+1, −*y*+1, −*z*+2; (ii) *x*+1, *y*, *z*; (iii) −*x*+2, −*y*+1, −*z*+1; (iv) −*x*+2, −*y*+2, −*z*+1; (v) −*x*, −*y*+1, −*z*+2; (vi) −*x*+1, −*y*+2, −*z*+1.

### (II) Crystal data

**Table:**

C_14_H_8_F_4_N_2_O_3_	*F*(000) = 664
*M**_r_* = 328.22	*D*_x_ = 1.672 Mg m^−^^3^
Monoclinic, *P*2_1_/*n*	Mo *K*α radiation, λ = 0.71073 Å
*a* = 12.285 (6) Å	Cell parameters from 5752 reflections
*b* = 5.945 (3) Å	θ = 3.3–30.5°
*c* = 17.888 (8) Å	µ = 0.16 mm^−^^1^
β = 93.44 (3)°	*T* = 100 K
*V* = 1304.1 (10) Å^3^	Rectangular prism, clear colourless
*Z* = 4	0.55 × 0.40 × 0.36 mm

### (II) Data collection

**Table:**

Bruker SMART APEX CCD diffractometer	3823 independent reflections
Radiation source: fine focus sealed tube	3443 reflections with *I* > 2σ(*I*)
Graphite monochromator	*R*_int_ = 0.025
Detector resolution: 8.3333 pixels mm^-1^	θ_max_ = 30.0°, θ_min_ = 2.0°
ω Scans scans	*h* = −16→17
Absorption correction: multi-scan (SADABS; Bruker, 2017)	*k* = −8→8
*T*_min_ = 0.80, *T*_max_ = 0.95	*l* = −25→25
18507 measured reflections	

### (II) Refinement

**Table:**

Refinement on *F*^2^	Primary atom site location: structure-invariant direct methods
Least-squares matrix: full	Hydrogen site location: inferred from neighbouring sites
*R*[*F*^2^ > 2σ(*F*^2^)] = 0.038	H-atom parameters constrained
*wR*(*F*^2^) = 0.105	*w* = 1/[σ^2^(*F*_o_^2^) + (0.0563*P*)^2^ + 0.5528*P*] where *P* = (*F*_o_^2^ + 2*F*_c_^2^)/3
*S* = 1.04	(Δ/σ)_max_ = 0.001
3823 reflections	Δρ_max_ = 0.47 e Å^−^^3^
208 parameters	Δρ_min_ = −0.25 e Å^−^^3^
0 restraints	

### (II) Special details

**Table:**

Geometry. All esds (except the esd in the dihedral angle between two l.s. planes) are estimated using the full covariance matrix. The cell esds are taken into account individually in the estimation of esds in distances, angles and torsion angles; correlations between esds in cell parameters are only used when they are defined by crystal symmetry. An approximate (isotropic) treatment of cell esds is used for estimating esds involving l.s. planes.

### (II) Fractional atomic coordinates and isotropic or equivalent isotropic displacement parameters (Å^2^)

**Table:**

	*x*	*y*	*z*	*U*_iso_*/*U*_eq_	
F1	0.61173 (7)	0.22134 (14)	0.90365 (4)	0.03327 (19)	
F2	0.48454 (5)	0.59673 (13)	0.88542 (4)	0.02357 (16)	
F3	0.59308 (6)	0.63688 (13)	0.63887 (4)	0.02478 (16)	
F4	0.71117 (6)	0.25516 (14)	0.66738 (4)	0.02993 (18)	
O1	0.47406 (6)	0.82103 (13)	0.75548 (4)	0.01844 (16)	
O2	0.30179 (7)	0.51138 (14)	0.73480 (4)	0.02086 (17)	
O3	0.45672 (7)	1.14224 (14)	0.64230 (5)	0.02420 (18)	
N1	0.66094 (8)	0.24215 (17)	0.78498 (6)	0.0230 (2)	
N2	0.39641 (7)	0.81650 (15)	0.69490 (5)	0.01503 (17)	
C1	0.60565 (9)	0.3286 (2)	0.83821 (6)	0.0215 (2)	
C2	0.54134 (8)	0.51817 (19)	0.82990 (6)	0.0178 (2)	
C3	0.53515 (8)	0.62643 (17)	0.76138 (6)	0.01569 (19)	
C4	0.59438 (8)	0.53864 (18)	0.70529 (6)	0.0178 (2)	
C5	0.65454 (9)	0.3444 (2)	0.72060 (6)	0.0208 (2)	
C6	0.30836 (8)	0.66728 (17)	0.69255 (6)	0.01506 (18)	
C7	0.23148 (8)	0.74245 (16)	0.62858 (5)	0.01440 (18)	
H7	0.156899	0.76892	0.646338	0.017*	
C8	0.22529 (9)	0.59170 (17)	0.55743 (6)	0.01733 (19)	
H8	0.178143	0.454959	0.559446	0.021*	
C9	0.34224 (9)	0.55197 (19)	0.53633 (6)	0.0201 (2)	
H9	0.381115	0.413986	0.540679	0.024*	
C10	0.38003 (9)	0.74523 (19)	0.51076 (6)	0.0204 (2)	
H10	0.450329	0.769546	0.49285	0.024*	
C11	0.29036 (9)	0.92015 (18)	0.51536 (6)	0.01784 (19)	
H11	0.296331	1.055951	0.482876	0.021*	
C12	0.28051 (8)	0.96515 (17)	0.60041 (6)	0.01497 (18)	
H12	0.230305	1.094276	0.608299	0.018*	
C13	0.38855 (8)	0.99715 (17)	0.64552 (6)	0.01595 (19)	
C14	0.18776 (9)	0.77180 (18)	0.49978 (6)	0.0193 (2)	
H14A	0.119519	0.847875	0.512318	0.023*	
H14B	0.180981	0.713441	0.447871	0.023*	

### (II) Atomic displacement parameters (Å^2^)

**Table:**

	*U*^11^	*U*^22^	*U*^33^	*U*^12^	*U*^13^	*U*^23^
F1	0.0372 (4)	0.0362 (4)	0.0258 (4)	0.0035 (3)	−0.0029 (3)	0.0160 (3)
F2	0.0194 (3)	0.0364 (4)	0.0149 (3)	−0.0003 (3)	0.0015 (2)	0.0005 (3)
F3	0.0241 (3)	0.0353 (4)	0.0149 (3)	0.0052 (3)	0.0016 (2)	0.0048 (3)
F4	0.0248 (4)	0.0381 (4)	0.0268 (4)	0.0120 (3)	0.0013 (3)	−0.0068 (3)
O1	0.0189 (3)	0.0198 (4)	0.0157 (3)	0.0032 (3)	−0.0064 (3)	−0.0024 (3)
O2	0.0214 (4)	0.0224 (4)	0.0189 (4)	−0.0009 (3)	0.0014 (3)	0.0074 (3)
O3	0.0243 (4)	0.0187 (4)	0.0289 (4)	−0.0060 (3)	−0.0042 (3)	0.0032 (3)
N1	0.0184 (4)	0.0233 (5)	0.0265 (5)	0.0023 (3)	−0.0049 (4)	0.0021 (4)
N2	0.0135 (4)	0.0177 (4)	0.0134 (4)	−0.0001 (3)	−0.0031 (3)	0.0017 (3)
C1	0.0195 (5)	0.0242 (5)	0.0202 (5)	−0.0011 (4)	−0.0039 (4)	0.0067 (4)
C2	0.0139 (4)	0.0242 (5)	0.0150 (4)	−0.0023 (4)	−0.0013 (3)	0.0013 (4)
C3	0.0122 (4)	0.0182 (4)	0.0162 (4)	−0.0008 (3)	−0.0029 (3)	0.0006 (3)
C4	0.0151 (4)	0.0231 (5)	0.0148 (4)	0.0003 (4)	−0.0018 (3)	0.0016 (4)
C5	0.0151 (4)	0.0248 (5)	0.0220 (5)	0.0024 (4)	−0.0020 (4)	−0.0032 (4)
C6	0.0138 (4)	0.0170 (4)	0.0146 (4)	0.0005 (3)	0.0021 (3)	0.0000 (3)
C7	0.0135 (4)	0.0155 (4)	0.0141 (4)	0.0004 (3)	−0.0003 (3)	0.0010 (3)
C8	0.0197 (5)	0.0151 (4)	0.0168 (4)	−0.0006 (3)	−0.0020 (3)	−0.0007 (3)
C9	0.0235 (5)	0.0196 (5)	0.0172 (5)	0.0056 (4)	0.0004 (4)	−0.0035 (4)
C10	0.0207 (5)	0.0256 (5)	0.0152 (5)	0.0027 (4)	0.0031 (4)	−0.0012 (4)
C11	0.0217 (5)	0.0180 (4)	0.0136 (4)	0.0009 (4)	−0.0006 (3)	0.0027 (3)
C12	0.0153 (4)	0.0140 (4)	0.0153 (4)	0.0014 (3)	−0.0014 (3)	0.0011 (3)
C13	0.0180 (4)	0.0141 (4)	0.0156 (4)	0.0019 (3)	−0.0001 (3)	−0.0002 (3)
C14	0.0209 (5)	0.0209 (5)	0.0155 (4)	0.0015 (4)	−0.0042 (4)	0.0000 (4)

### (II) Geometric parameters (Å, º)

**Table:**

F1—C1	1.3312 (14)	C7—C12	1.5511 (15)
F2—C2	1.3323 (13)	C7—C8	1.5547 (15)
F3—C4	1.3231 (13)	C7—H7	1.0
F4—C5	1.3234 (13)	C8—C9	1.5256 (16)
O1—C3	1.3796 (13)	C8—C14	1.5380 (15)
O1—N2	1.4004 (12)	C8—H8	1.0
O2—C6	1.2017 (13)	C9—C10	1.3305 (17)
O3—C13	1.2060 (14)	C9—H9	0.95
N1—C5	1.3005 (16)	C10—C11	1.5207 (16)
N1—C1	1.3076 (16)	C10—H10	0.95
N2—C13	1.3901 (14)	C11—C14	1.5499 (16)
N2—C6	1.3975 (14)	C11—C12	1.5565 (16)
C1—C2	1.3791 (16)	C11—H11	1.0
C2—C3	1.3824 (15)	C12—C13	1.5233 (15)
C3—C4	1.3769 (15)	C12—H12	1.0
C4—C5	1.3891 (16)	C14—H14A	0.99
C6—C7	1.5071 (15)	C14—H14B	0.99
			
C3—O1—N2	112.66 (8)	C14—C8—C7	98.38 (9)
C5—N1—C1	117.04 (10)	C9—C8—H8	116.0
C13—N2—C6	116.56 (9)	C14—C8—H8	116.0
C13—N2—O1	119.66 (8)	C7—C8—H8	116.0
C6—N2—O1	121.70 (8)	C10—C9—C8	107.73 (9)
N1—C1—F1	116.61 (11)	C10—C9—H9	126.1
N1—C1—C2	124.18 (10)	C8—C9—H9	126.1
F1—C1—C2	119.20 (11)	C9—C10—C11	107.64 (10)
F2—C2—C1	121.89 (10)	C9—C10—H10	126.2
F2—C2—C3	119.63 (10)	C11—C10—H10	126.2
C1—C2—C3	118.47 (10)	C10—C11—C14	100.67 (9)
C4—C3—O1	124.82 (9)	C10—C11—C12	105.64 (8)
C4—C3—C2	117.79 (10)	C14—C11—C12	99.41 (8)
O1—C3—C2	117.32 (9)	C10—C11—H11	116.2
F3—C4—C3	120.56 (10)	C14—C11—H11	116.2
F3—C4—C5	121.43 (10)	C12—C11—H11	116.2
C3—C4—C5	118.00 (10)	C13—C12—C7	106.11 (8)
N1—C5—F4	116.30 (10)	C13—C12—C11	115.01 (9)
N1—C5—C4	124.49 (10)	C7—C12—C11	103.02 (8)
F4—C5—C4	119.20 (10)	C13—C12—H12	110.8
O2—C6—N2	123.48 (10)	C7—C12—H12	110.8
O2—C6—C7	130.06 (10)	C11—C12—H12	110.8
N2—C6—C7	106.45 (9)	O3—C13—N2	124.12 (10)
C6—C7—C12	105.18 (8)	O3—C13—C12	130.51 (10)
C6—C7—C8	116.63 (9)	N2—C13—C12	105.38 (8)
C12—C7—C8	103.12 (8)	C8—C14—C11	93.88 (8)
C6—C7—H7	110.5	C8—C14—H14A	112.9
C12—C7—H7	110.5	C11—C14—H14A	112.9
C8—C7—H7	110.5	C8—C14—H14B	112.9
C9—C8—C14	101.00 (9)	C11—C14—H14B	112.9
C9—C8—C7	106.93 (8)	H14A—C14—H14B	110.4
			
C3—O1—N2—C13	−130.66 (10)	N2—C6—C7—C8	107.89 (10)
C3—O1—N2—C6	66.28 (12)	C6—C7—C8—C9	−50.39 (11)
C5—N1—C1—F1	179.27 (10)	C12—C7—C8—C9	64.30 (10)
C5—N1—C1—C2	0.48 (17)	C6—C7—C8—C14	−154.65 (9)
N1—C1—C2—F2	178.42 (10)	C12—C7—C8—C14	−39.97 (9)
F1—C1—C2—F2	−0.34 (16)	C14—C8—C9—C10	31.98 (11)
N1—C1—C2—C3	−0.67 (17)	C7—C8—C9—C10	−70.39 (11)
F1—C1—C2—C3	−179.43 (10)	C8—C9—C10—C11	1.02 (12)
N2—O1—C3—C4	55.79 (13)	C9—C10—C11—C14	−33.35 (11)
N2—O1—C3—C2	−127.50 (10)	C9—C10—C11—C12	69.69 (11)
F2—C2—C3—C4	−179.44 (9)	C6—C7—C12—C13	4.58 (10)
C1—C2—C3—C4	−0.33 (15)	C8—C7—C12—C13	−118.12 (9)
F2—C2—C3—O1	3.61 (14)	C6—C7—C12—C11	125.81 (8)
C1—C2—C3—O1	−177.28 (9)	C8—C7—C12—C11	3.12 (9)
O1—C3—C4—F3	−2.24 (16)	C10—C11—C12—C13	45.57 (11)
C2—C3—C4—F3	−178.94 (9)	C14—C11—C12—C13	149.54 (9)
O1—C3—C4—C5	178.10 (9)	C10—C11—C12—C7	−69.40 (10)
C2—C3—C4—C5	1.40 (15)	C14—C11—C12—C7	34.56 (9)
C1—N1—C5—F4	179.84 (10)	C6—N2—C13—O3	178.19 (10)
C1—N1—C5—C4	0.74 (17)	O1—N2—C13—O3	14.27 (15)
F3—C4—C5—N1	178.64 (10)	C6—N2—C13—C12	−1.96 (12)
C3—C4—C5—N1	−1.70 (17)	O1—N2—C13—C12	−165.88 (8)
F3—C4—C5—F4	−0.44 (16)	C7—C12—C13—O3	177.99 (11)
C3—C4—C5—F4	179.22 (10)	C11—C12—C13—O3	64.81 (15)
C13—N2—C6—O2	−175.80 (10)	C7—C12—C13—N2	−1.85 (10)
O1—N2—C6—O2	−12.23 (15)	C11—C12—C13—N2	−115.02 (9)
C13—N2—C6—C7	5.00 (12)	C9—C8—C14—C11	−48.57 (9)
O1—N2—C6—C7	168.57 (8)	C7—C8—C14—C11	60.61 (9)
O2—C6—C7—C12	175.23 (11)	C10—C11—C14—C8	49.16 (9)
N2—C6—C7—C12	−5.64 (10)	C12—C11—C14—C8	−58.86 (9)
O2—C6—C7—C8	−71.24 (14)		
